# Knowledge and self-care practices regarding diabetes among newly diagnosed type 2 diabetics in Bangladesh: a cross-sectional study

**DOI:** 10.1186/1471-2458-12-1112

**Published:** 2012-12-26

**Authors:** Farzana Saleh, Shirin J Mumu, Ferdous Ara, Housne A Begum, Liaquat Ali

**Affiliations:** 1 Department of Community Nutrition, Bangladesh Institute of Health Sciences (BIHS), Dhaka, Bangladesh; 2 Department of Epidemiology, Bangladesh Institute of Health Sciences (BIHS), Dhaka, Bangladesh; 3 Institute of Health Economics, University of Dhaka, Dhaka, Bangladesh; 4 Department of Biochemistry and Cell Biology, Bangladesh Institute of Health Sciences (BIHS), Dhaka, Bangladesh

**Keywords:** Bangladesh, Type 2 diabetes, Knowledge, Self-care, Practice, Diabetes

## Abstract

**Background:**

Levels of knowledge about diabetes mellitus (DM) among newly diagnosed diabetics in Bangladesh are unknown. This study assessed the relationship between knowledge and practices among newly diagnosed type 2 DM patients.

**Methods:**

Newly diagnosed adults with type 2 diabetes (N = 508) were selected from 19 healthcare centers. Patients’ knowledge and self-care practices were assessed via interviewer-administered questionnaires using a cross-sectional design. Knowledge questions were divided into basic and technical sections. Knowledge scores were categorized as poor (<mean – 1 SD), average (mean ± 1 SD), good (>mean + 1 SD). Chi square testing and multivariate logistic regression were conducted to examine the relationship between diabetes-related knowledge and self-care practices.

**Results:**

Approximately 16%, 66%, and 18% of respondents had good, average, and poor (GAP) basic knowledge respectively and 10%, 78%, and 12% of respondents had GAP technical knowledge, about DM. About 90% of respondents from both basic and technical GAP did not test their blood glucose regularly; a significant relationship existed between basic knowledge and glucose monitoring. Technical knowledge and foot care were significantly related, though 81% with good technical knowledge and about 70% from average and poor groups did not take care of their feet. Approximately 85%, 71%, and 52% of the GAP technical knowledge groups, consumed betel nuts; a significant relationship existed between technical knowledge and consumption of betel nuts. Around 88%, 92%, and 98% of GAP technical knowledge groups failed to follow dietary advice from a diabetes educator. About 26%, 42%, and 51% of GAP basic and technical sometimes ate meals at a fixed time (p < 0.05). Approximately one-third of respondents in each basic knowledge group and 29%, 32%, and 32% of GAP technical knowledge groups partially followed rules for measuring food before eating. Total basic knowledge (TBK) and business profession were significant independent predictors of good practice. OR for TBK: 1.28 (95% CI: 1.03 to 1.60); OR for business profession 9.05 (95% CI: 1.17 to 70.09).

**Conclusions:**

Newly diagnosed type 2 diabetics had similar levels of basic and technical knowledge of DM. Health education and motivation should create positive changes in diabetes-control-related self-care practices.

## Background

Diabetes mellitus (DM) is a major disease that is becoming more prevalent, affecting more than 171 million people worldwide. The number of people affected by DM is expected to rise to 366 million by 2030 [1]. Demographic transition, combined with urbanization and industrialization, has resulted in drastic changes in lifestyles globally. Consequently, lifestyle-related diseases like DM have emerged as major public health problems. Diabetes is characterized by a state of chronic hyperglycemia resulting from several environmental and genetic etiologies acting jointly [2]. Until a decade ago, diabetes was not considered a major public health problem in developing countries like Bangladesh, but the situation has now changed dramatically. According to the International Diabetes Federation (IDF) report (2011), Bangladesh now leads the world with 8.4 million diabetic patients, and this number is projected to increase to 16.8 million by the year 2030 [3]. In Bangladesh, a higher prevalence of diabetes was found in urban (8.1%) compared with rural (2.3%) populations [4].

Diabetes is a silent disease: many sufferers become aware that they have diabetes only when they develop one of its life-threatening complications [5]. Knowledge of diabetes mellitus can assist in early detection of the disease and reduce the incidence of complications. Levels of knowledge about diabetes among the at-risk population and among those who suffer from the disease are unknown, but more knowledge is associated with better outcomes.

There have been few studies on knowledge about diabetes among newly diagnosed diabetic patients in developing countries like Bangladesh, but studies such as these are crucial for the appropriate use of limited resources in poor socioeconomic and educational conditions. The objective of this study was to test the relationship between knowledge and self-care practices among newly diagnosed type 2 diabetic subjects.

## Methods

A cross-sectional study design was adopted, and 508 newly diagnosed type 2 diabetic patients were selected conveniently in consideration of the inclusion and exclusion criteria from 19 healthcare centers. The minimum required sample size from each center was calculated using the formula [6]*n* = 15.4  ×  *p*  ×  (1 − *q*)/*w*^2^ (where n = the required sample size, p = the expected proportion, and w = width of the confidence interval), and patients were selected from the healthcare centers’ last month of patient records. Patients who had other medical complications or were unable to answer a short list of simple questions (sociodemographic information such as name, address, disease complications, etc.) were excluded from the study.

A method that has been used in various studies in different countries [7-9] was adapted for this study of knowledge and self-care practices in a Bangladeshi population. The knowledge and self-care practices of the subjects were assessed via an interviewer-administered questionnaire, and the interview was administered in an outpatient department (OPD) setting. A medium-sized–three-part questionnaire was designed by the researcher. The first part of the questionnaire consisted of sociodemographic information, family history of diabetes, anthropometric measurements, and clinical and biochemical reports. Part two consisted of 35 knowledge questions, and part three focused on steps taken to monitor glucose, control calorie and food intake, exercise, practice foot care, and take other actions indicative of patient lifestyle. The Diabetes Knowledge Test (DKT) questionnaire, which was validated by the University of Michigan [10], was modified and used for data collection. This questionnaire was translated to Bangla by two separate translators who were native speakers of the target language (Bangla); two separate back-translations were done by translators who were native speakers of English. Knowledge questions were also substantively modified according to the local guidelines of the Diabetic Association of Bangladesh [11]. The knowledge assessment questionnaire included questions about diabetes, blood testing, hyperglycemia, and general principles of disease care. A pre-test was conducted before the questionnaire was finalized.

During analysis, knowledge questions were divided into basic and technical sections; 13 items were included in the basic part, which consisted of fundamental knowledge of diabetes. Twenty-two technical knowledge questions involved such concepts as the target age for diabetes testing, the benefits of exercise, hyperglycemia, groupings of foods and their exchange list, ideal body weight, and ketoacidosis. Each correct response was assigned a score of 1, and each incorrect response was assigned a score of 0. Thus, for 13 items for basic knowledge, the maximum attainable score was 13 and the minimum score was 0. For 22 technical knowledge items, the maximum attainable score was 22 and minimum was 0. Similarly, for eight practice item such as glucose monitoring, exercise, foot care, smoking, consumption of betel nuts, groupings of foods and their exchange list, the maximum attainable score was 8 and minimum was 0. The level of basic, technical knowledge and practice was classified according to each respondent’s score. Poor knowledge and practice corresponded to a score of (<Mean – 1 SD); average knowledge and practice corresponded to a score between (Mean ± 1 SD); good knowledge and practice corresponded to a score of (>Mean + 1 SD) [12]. Statistical tests were considered significant at p-values ≤5% (≤0.05). Frequencies were calculated for descriptive analysis. Chi-squared tests were performed on categorical data to find the relationships between variables. Multivariate logistic regression was performed to identify the predictors of self-care practices. Socioeconomic classifications in this study were made according to the 2006 per capita Gross National Income (GNI) and according to World Bank (WB) calculations [13]. The groups were: low-income, US$ ≤ 905 or Bangladeshi Taka; BDT ≤5360; lower-middle-income, US$ (906–3595) or BDT (5361–21270); upper-middle-income, US$ (3596–11115) or BDT (21271–65761); and high-income, US$ ≥11116 or BDT ≥65762. Informed written consent was obtained from all respondents after a full explanation of the nature, purpose, and procedures used for the study. Ethical approval was obtained from the ethics and research review committees of the Diabetic Association of Bangladesh.

## Results

Mean age of the respondents was 45.0 ± 9.5 years; about half of them (53%) were female. About 20% were illiterate, 20% had a primary through 8^th^-grade education, 30% had secondary education, and only 17% had graduated from college. About half of the respondents (47%) lived in urban areas, and the rest lived in semi-urban and rural areas. Half of the respondents (48%) were homemakers, and others were service providers (15%), businesspeople (23%), or either unemployed or laborers (14%). Slightly more than half of the respondents (55%) belonged to the lower-middle-income group, one-fourth belonged to the low-income group, approximately 20% belonged to the upper-middle-income group, and only 5% belonged to the high-income group (Table 1).

**Table 1:** Characteristics of the study subjects (N = 508)

**Parameters**	
Age, years	44.96 ± 9.5
Gender	
Male	240 (47%)
Female	268 (53%)
Education	
Illiterate	101 (20%)
Primary to 8^th^ grade	165 (33%)
Secondary–Higher Secondary	152 (30%)
Graduate & above	90 (17%)
Habitat	
Urban	237 (47%)
Semi-urban	109 (22%)
Rural	162 (31%)
Occupation	
Service	76 (15%)
Business	119 (23%)
Homemaker	245 (48%)
Others (laborer/unemployed)	68 (14%)
Monthly Income (US$)	
Low income (≤905)	120 (24%)
Lower-middle income (906–3595)	277 (55%)
Upper-middle income (3596–11115)	88 (17%)
High income ( ≥11116)	23 (5%)

Results are expressed as number (%) and mean ± SD; US$1 = 80 Bangladeshi Taka (BDT).

**Figure 1:**
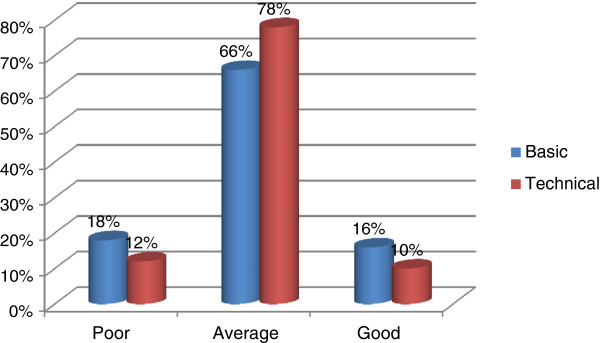
Distribution of study subjects according to the level of knowledge regarding DM (N = 508).

The knowledge distribution of the subjects regarding fundamental components of diabetes management is shown in Figure 1. The mean basic knowledge score of the respondents was 6 ±3. Approximately 66% of respondents had average (3–9) basic knowledge regarding diabetes. Only about 18% had poor (<3) knowledge regarding fundamental aspects of DM, and 16% had good (>9) knowledge. Regarding technical knowledge, mean score among respondents was 12±4. Majority of the respondents (78%) had average (8–16) knowledge, and 12% had poor (<8) knowledge. Only 10% had good (>16) technical knowledge of DM.

Table 2 shows the summary results of Chi-square (χ^2^) analysis between level of total basic & technical knowledge and different types of self-care practices among the respondents. A significant relationship existed between basic knowledge and glucose monitoring, though, about 90% of respondents in each knowledge group did not test their blood glucose regularly. About 81%, 72%, and 52% of subjects with good, average, and poor (GAP) knowledge, respectively, did exercise, and the relationship was significant. The relationship between the level of knowledge and foot care was not significant, and more than 70% respondents did not take care of their feet in all three knowledge groups. Approximately 80% of patients in each knowledge group smoked regularly, and 83%, 69%, and 63% of subjects in GAP knowledge groups consumed betel nuts, a significant relationship.

**Table 2:** Summary results of χ^2^ analysis between level of total basic & technical knowledge Vs different types of practices (N = 508)

***Level of Knowledge***	***X***^***2***^	***d.f***	***p***
a.** Basic Knowledge (Good, average, poor)**	25.35	2	0.0001
x BG testing (yes/no)	20.11	2	0.0001
x Doing Exercise (yes/no)	0.81	2	0.67
x foot care (yes/no)	0.61	2	0.74
x smoking (yes/no)	8.51	2	0.01
x consumption of Betel nuts (yes/no)	5.16	4	0.30
x followed dietary advice given by diabetes educator (FF/PF/NF)	16.66	4	0.002
x followed fixed time for eating main meal (FF/PF/NF)	36.26	4	0.0001
x measured food before eating meal (FF/PF/NF)			
b. **Technical Knowledge (Good, average, poor)**	1.28	2	0.50
x BG testing (yes/no)	23.20	2	0.0001
x Doing Exercise (yes/no)	1.80	2	0.41
x foot care (yes/no)	0.93	2	0.60
x smoking (yes/no)	15.11	2	0.001
x consumption of Betel nuts (yes/no)	10.87	4	0.03
x followed dietary advice given by diabetes educator (FF/PF/NF)	10.74	4	0.03
x followed fixed time for eating main meal (FF/PF/NF)	16.01	4	0.003
x measured food before eating meal (FF/PF/NF)			

FF=Fully Followed; PF= Partially Followed; NF= Not Followed.

More than 90% of the respondents from good (92%), average (91%), and poor (97%) basic knowledge groups did not follow the dietary advice given by diabetes educator. About 26%, 42%, and 51% of GAP basic knowledge groups partially observed fixed times for eating their main meals, a significant relationship. Approximately one-third of respondents in each knowledge group partially followed the rules for measuring food before eating, a significant relationship.

About 90% of respondents from all three technical knowledge groups did not test their blood glucose regularly, and the relationship was not significant. About 85%, 72%, and 46% of GAP technical knowledge groups exercised regularly, a significant relationship. The relationship between technical knowledge and foot care was not significant and 81% from the good technical knowledge group did not take care of their feet. Respondents in the average (74%) and poor (79%) technical knowledge groups also tended not to take care of their feet. Eight of ten respondents smoked regularly in each technical knowledge group, and 85%, 71%, and 52% of GAP technical knowledge groups consumed betel nuts; the relationship between betel nut consumption and level of technical knowledge was significant.

About 90% of respondents from the good (88%), average (92%), and poor (98%) technical knowledge groups did not follow dietary advice given by a diabetes educator. The rest of the respondents from each knowledge group either fully followed or partially followed the advice, a significant relationship. Around 23% of the respondents from the good, average (41%), and poor (51%) knowledge groups partially observed fixed times for eating their main meals, a significant relationship. Approximately one-third of the respondents from the good (29%), average (32%), and poor (32%) knowledge groups partially followed the rules for measuring food before eating, a significant relationship.

Results of the multivariate logistic regression analysis are presented in Table 3. The mean practice score of the respondents was 3 ± 1. The level of practice was classified as per each respondent’s score viz. <2 score as ‘poor’; 2–4 as ‘average’ and >4 as ‘good’. In model 1, total basic knowledge (TBK) and business profession were significant independent predictors of good practice. OR for TBK: 1.27 (95% CI: 1.03 to 1.60); OR for business profession 9.05 (95% CI: 1.17 to 70.08). Total technical knowledge (TTK) also tended as an independent predictor (of good practice) with an odds ratio of 1.16 (95% CI: 0.99 to 1.37). In the second model high income group was negatively associated with average practice, with an odds ratio of 0.15 (95% CI: 0.03 to 0.77). TBK and TTK did not play any significant role in this model.

**Table 3:** Multinomial logistic regression for estimating the odds ratio and 95% confidence interval for practicing good and average (with poor practice as the reference category) by the selected factors

**Level of Practice**	**Independent variables**	** ß**	**Sig.**	**Odds ratio**	**95% C.I. for EXP(ß)**	
					** Lower**	**Upper**
Good	**Age (years)**					
	<40	−0.65	0.19	0.52	0.19	1.39
	>40	Reference				
	**Monthly income (BDT)**					
	High income group	−1.15	0.25	0.32	0.04	2.22
	Lower middle income group	−0.37	0.53	0.69	0.22	2.20
	Upper middle income group	−1.21	0.12	0.29	0.06	1.35
	Low income group	Reference				
	**Gender**					
	Male	0.26	0.76	1.29	0.23	7.24
	Female	Reference				
	**Total Basic knowledge**	0.25	0.03	1.28	1.03	1.60
	**Total Technical knowledge**	0.15	0.06	1.16	0.99	1.37
	**Occupation**					
	Service	−0.40	0.60	0.66	0.14	3.07
	Homemaker	−0.46	0.50	0.62	0.16	2.44
	Business	2.20	0.03	9.05	1.17	70.08
	Others (unemployed)	Reference				
	**Level of Education**					
	Primary to 8^th^ grade	0.81	0.24	2.24	0.58	8.65
	Secondary–Higher Secondary	1.10	0.15	3.00	0.65	13.80
	Graduate & above	1.27	0.15	3.56	0.61	20.66
	Illiterate	Reference				
	**Habitat**					
	Urban	0.55	0.33	1.74	0.56	5.33
	Semi urban	0.06	0.92	1.06	0.29	3.83
	Rural	Reference				
	**Family history of diabetes**					
	Yes	0.11	0.81	1.12	0.42	2.96
	No	Reference				
	**Information from inside health care center**					
	Yes	0.62	0.23	1.86	0.67	5.19
	No	Reference				
	**Information from outside health care center**					
	Yes	0.21	0.73	1.23	0.37	4.01
	No	Reference				
**Average**	**Age (years)**					
	<40	−0.25	0.51	0.77	0.35	1.68
	>40	Reference				
	**Monthly income (BDT)**					
	High income group	−1.85	0.02	0.15	0.03	0.77
	Lower middle income group	−0.28	0.51	0.75	0.32	1.75
	Upper middle income group	−0.89	0.12	0.40	0.12	1.29
	Low income group	Reference				
	**Gender**					
	Male	−0.33	0.64	0.71	0.17	2.95
	Female	Reference				
	**Total Basic knowledge**	0.12	0.15	1.13	0.95	1.34
	**Total Technical knowledge**	0.09	0.12	1.09	0.97	1.22
	**Occupation**					
	Service	−0.31	0.60	0.73	0.22	2.36
	Homemaker	−0.28	0.57	0.75	0.27	2.04
	Business	1.17	0.16	3.25	0.60	17.40
	Others (unemployed)	Reference				
	**Level of Education**					
	Primary to 8^th^ grade	0.11	0.81	1.11	0.45	2.75
	Secondary–Higher Secondary	0.47	0.39	1.61	0.53	4.81
	Graduate & above	0.07	0.91	1.07	0.29	4.01
	Illiterate	Reference				
	**Habitat**					
	Urban	0.67	0.13	1.95	0.81	4.69
	Semi urban	0.71	0.14	2.04	0.78	5.32
	Rural	Reference				
	**Family history of diabetes**					
	Yes	0.16	0.65	1.17	0.56	2.43
	No	Reference				
	**Information from inside health care center**					
	Yes	−0.08	0.84	0.91	0.38	2.17
	No	Reference				
	**Information from outside health care center**					
	Yes	0.04	0.91	1.05	0 .45	2.44
	No	Reference				

ß for standardized regression coefficient. Level of practice was taken as dependent variable whereas other variables were taken as independent variables.

## Discussion

The available scientific knowledge concerning diabetes mellitus is an important resource to guide and educate diabetes patients concerning self-care. Self-care concepts that can benefit patients include adherence to diet, physical activity, blood glucose monitoring, and taking oral medication and insulin. Few studies regarding the relationship between knowledge and self-care practices among newly diagnosed diabetics are available in Bangladesh or elsewhere in the world. Studies have mostly involved the general population and type 2 diabetes patients who have had the disease for a significant period of time [5,12,14-17]. This study was undertaken in order to assess the relationships between knowledge and self-care practices among newly diagnosed type 2 diabetics attending different healthcare centers in Bangladesh.

In the present study, it is encouraging to note that the majority of respondents had average basic (66%) and technical (78%) knowledge regarding diabetes mellitus (DM). A study was conducted on members of the general public in Singapore to evaluate their level of knowledge about diabetes, and the results indicated that the respondents had an acceptable level of knowledge [5]. Another study was done on knowledge and perceptions of diabetes in a semi-urban Omani population; it found that subjects’ level of knowledge was suboptimal [14]. A study conducted on people with diabetes attending the Aga Khan University Hospital (AKUH) [15] in Pakistan found that 12%, 35%, and 53% of the patients had GAP knowledge of the symptoms, treatments, and complications of diabetes.

With regard to self-care practices, it was unfortunate to note that the majority (90%) of this study’s respondents from all three basic knowledge groups did not test their blood glucose regularly. However, the relationship was significant. Similar results were found in technical knowledge groups, and the relationship was not significant. These results revealed that the frequency of blood glucose monitoring increases gradually as the level of knowledge changes. The patients in this study showed higher rates of self-monitoring than those found in the study from Singapore [16].

Further findings indicated that a good number of the respondents in each basic knowledge group did exercise, and the rate of exercise rose with increasing levels of knowledge. Similar and significant results were found in this study’s technical knowledge groups. In Peshawar, it was found that 75% of subjects who had had diabetes for 9 years did exercise in order to control blood glucose [17]. In the present study, many respondents in all three basic and technical knowledge groups did not take extra care of their feet regularly. Moreover, only 16%, 13%, and 12% of patients in GAP basic knowledge groups did not smoke. Almost the same rates of smoking were found in the technical knowledge groups. About 80% of respondents with good levels of basic knowledge consumed betel nuts; more than half of the respondents from the poor and average basic knowledge groups also had the same practice. Similar results were found in the three technical knowledge groups, and the relationship was significant both in basic and technical knowledge groups.

Diet plays an important role in the prevention and management of DM. The majority (90%) of the respondents in each basic and technical knowledge group did not follow dietary advice given by diabetes educators. Diabetes significantly changes the relationships between patients, their bodies, and the world around them, and restrictions on eating habits make them more aware of their limitations. This is why the conflict between the desire to eat and the imperious need to refrain from indulging such desire is always present in the daily lives of people with diabetes. This conflict might be an important element in understanding the respondents’ low rates of positive practices for coping with the disease. About 26%, 42%, and 51% of GAP basic knowledge groups were aware of the practice of following fixed times for eating their main meals, a significant relationship. Similar and significant results were found in the technical knowledge groups. Notably, about one-third of respondents in all basic and technical knowledge groups partially practiced the measurement of food before eating, a significant result.

Respondents of the present study were fairly informed about diabetes management and we have found an association between basic knowledge and practice. There is evidence that patient awareness is the most effective way to lessen the complications of diabetes [18]. Business, one of the categories of occupations, has also been identified as determinant of good practice. We assume that this might have been due to their better access to goods and services as well their independence in availing the health care. Contrarily, rich people showed lower level of practice. The reason needs exploration.

In this study, several explanations were possible for the fact that respondents had average knowledge of DM but inappropriate self-care practices. First, the bulk of the respondents had family history of diabetes. It would be reasonable to assume that diabetic family members would share their knowledge with non-diabetics and newly diagnosed diabetics. Second, as the respondents in this study were newly diagnosed, they had not attended any structured diabetes education programs. Ignorance, high confidence level and lack of time may also be the reasons behind the scenario. Various issues need to be addressed in order to close the gaps between knowledge and practice. The results of this study encourage a positive outlook: all that is required is that a diabetes educator trained in diabetes management counsel patients during every visit and counseling may have an impact in improving the perception about disease, diet, and lifestyle changes and thereby on glycemic control and the complications of diabetes.

## Conclusions

In this study, newly diagnosed type 2 diabetic subjects had similar levels of both basic and technical knowledge of DM. Repeated reinforcement of health education and strong motivation are bound to bring about positive changes in self-care practices with regard to diabetes control.

## Competing interests

The authors declare that they have no competing interests.

## Authors’ contributions

FS: contributed her intellectual ability to conception and design of the research, analysis and interpretation of data; drafting the article, revising it critically for important intellectual content; and final approval of the version to be published. SJM: contributed her intellectual ability to conception and design of the research, analysis and interpretation of data; drafting the article and revising it critically for important intellectual content; and final approval of the version to be published. FA: contributed her intellectual ability to conception and design of the research, analysis and interpretation of data; drafting the article and revising it critically for important intellectual content; and final approval of the version to be published. HAB: Revision of manuscript for important intellectual content. LA: Revision of manuscript for important intellectual content. All of the above authors read and approved the final manuscript.

## Pre-publication history

The pre-publication history for this paper can be accessed here:

http://www.biomedcentral.com/1471-2458/12/1112/prepub
